# Tryptophan Catabolites in Bipolar Disorder: A Meta-Analysis

**DOI:** 10.3389/fimmu.2021.667179

**Published:** 2021-05-19

**Authors:** Kaat Hebbrecht, Katrien Skorobogatov, Erik J. Giltay, Violette Coppens, Livia De Picker, Manuel Morrens

**Affiliations:** ^1^ Faculty of Medicine and Health Sciences, Collaborative Antwerp Psychiatric Research Institute (CAPRI), University of Antwerp, Antwerp, Belgium; ^2^ Scientific Initiative of Neuropsychiatric and Psychopharmacological Studies (SINAPS), University Psychiatric Centre Duffel, Duffel, Belgium; ^3^ Department of Psychiatry, Leiden University Medical Center, Leiden, Netherlands

**Keywords:** bipolar disorder, inflammation, immune, kynurenine, tryptophan, depression

## Abstract

**Objective:**

Tryptophan catabolites (TRYCATs) are implicated in the pathophysiology of mood disorders by mediating immune-inflammation and neurodegenerative processes. We performed a meta-analysis of TRYCAT levels in bipolar disorder (BD) patients compared to healthy controls.

**Methods:**

A systematic literature search in seven electronic databases (PubMed, Embase, Web of Science, Cochrane, Emcare, PsycINFO, Academic Search Premier) was conducted on TRYCAT levels in cerebrospinal fluid or peripheral blood according to the PRISMA statement. A minimum of three studies per TRYCAT was required for inclusion. Standardized mean differences (SMD) were computed using random effect models. Subgroup analyses were performed for BD patients in a different mood state (depressed, manic). The methodological quality of the studies was rated using the modified Newcastle-Ottawa Quality assessment Scale.

**Results:**

Twenty-one eligible studies were identified. Peripheral levels of tryptophan (SMD = -0.44; *p* < 0.001), kynurenine (SMD = - 0.3; *p* = 0.001) and kynurenic acid (SMD = -.45; *p* = < 0.001) were lower in BD patients versus healthy controls. In the only three eligible studies investigating TRP in cerebrospinal fluid, tryptophan was not significantly different between BD and healthy controls. The methodological quality of the studies was moderate. Subgroup analyses revealed no significant difference in TRP and KYN values between manic and depressed BD patients, but these results were based on a limited number of studies.

**Conclusion:**

The TRYCAT pathway appears to be downregulated in BD patients. There is a need for more and high-quality studies of peripheral and central TRYCAT levels, preferably using longitudinal designs.

## Introduction

Bipolar disorder (BD) is a chronic psychiatric disorder characterized by alternating periods of depression and abnormally elevated moods. BD is one of the leading causes of global disability, resulting in cognitive and functional decline and an increased mortality rate (1). The pathophysiology of BD remains to be fully elucidated but accumulating evidence points towards a pathophysiological role of chronic low-grade inflammation (2).

**Figure 1:**
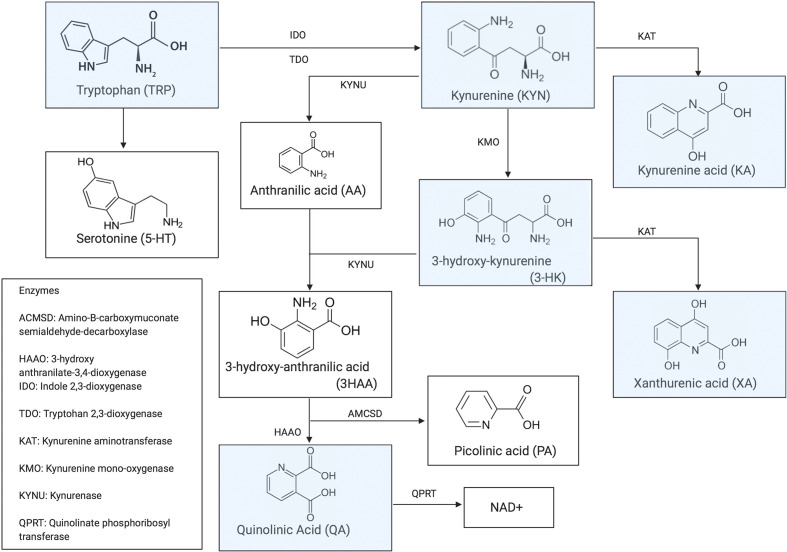
Kynurenine Pathway [previously published in Morrens et al. (4)].

The kynurenine pathway of tryptophan (TRP) degradation has been proposed as the missing link through which inflammation causes neurotoxicity and psychiatric symptoms. TRP is an essential amino acid and a precursor for serotonin or 5‐hydroxytryptamine. In response to inflammation or psychosocial stress (3), TRP is primarily metabolized into kynurenine (KYN) following an upregulation of indoleamine 2,3‐deoxygenase (IDO‐1) and hereby leading to a reduction in availability of serotonin (for a graphical illustration of the KYN Pathway, see **Figure 1**). This depletion of serotonin has been assumed to play a major role in the pathophysiology of depression (5, 6). More recent studies also point towards the imbalance supposedly neurotoxic [including 3-hydroxy kynurenine (3-HK) and quinolinic acid (QA)) and neuroprotective (kynurenic acid (KA)] TRP catabolites (TRYCAT) as a central mechanism in the pathophysiology of mood disorders (7, 8). In patients with Major Depressive Disorder (MDD), a consistent increase in 3-HK and QA and a decrease in KA in blood and cerebrospinal fluid has been found (8, 9). In BD patients, however, results have been more divergent and appear specific to the symptomatic state (10). In depressed or euthymic BD patients, TRYCAT alterations seem to be similar to those in MDD (11–13). In contrast, BD patients with a history of psychosis have shown elevated KA levels in cerebrospinal fluid (CSF) but not in the periphery, analogous to schizophrenia patients (13–16).

In the last decade, a growing number of studies in BD has been published and TRYCATs are represented as promising biomarkers related to BD (17). However, studies show conflicting results and there is a great variation in methodological quality between studies, with a potential risk of bias as a consequence. Two previous meta-analyses synthesized the role of kynurenine metabolites in broad psychiatric disorders (18, 19). Both included a limited number of studies in BD which investigated only a limited selection of TRYCATs (mostly TRP, KYN and/or KA) and the impact of mood state was not investigated. Arnone and colleagues (18) reported no significant differences in peripheral KYN or TRP values compared to controls, but only five studies were included and there was considerable heterogeneity among studies. The meta-analysis by Wang and Miller (19). found that CSF levels of KA were significantly increased in euthymic BD patients compared to healthy controls, but this finding were based upon two studies with partly overlapping samples (15, 16). A third, recently published, meta-analysis summarized the results of studies on TRYCATs in BD, but they included only studies investigating TRYCAT levels in peripheral blood that were published after 2006. Furthermore, they did not provide a critical evaluation of the study quality (20).

The aim of this meta-analysis is to synthesize the available evidence on peripheral and central TRYCAT alterations in case-control studies of BD patients and to critically evaluate the quality of available studies. Furthermore, subgroup analyses were performed to separately investigate the differences in TRYCAT levels in manic (BD-M) and bipolar depressed (BD-D) patients.

## Material and Methods

This meta-analysis was conducted and written according to the principles of the PRISMA-P (preferred reporting items for systematic review and meta-analysis protocols) guidelines (21).

### Search Strategy

A search of seven electronic databases (PubMed, Embase, Web of Science, Cochrane, Emcare, PsycINFO, Academic Search Premier) was conducted for original papers on levels of TRYCATs (i.e. TRP, KYN, KA, 3-HK, QA) in BD patients. A medical librarian of the University of Leiden was involved in the establishment of the search strings (see **Supplementary S1**) and the literature search (last search: August, 19, 2020). Two authors (M.M. and K.H.) independently assessed studies for suitability for inclusion.

Inclusion criteria for eligible papers were: 1) English language papers published in peer-reviewed journals; 2) Case-control studies comparing BD patients (as confirmed by Research Diagnostic Criteria (RDC), DSM-(III, III-R, IV, IV-TR) or ICD-(9 or 10) to healthy controls, 3) assessment of at least 1 TRYCAT metabolite in peripheral blood, CSF or postmortem tissue. In case of sample overlap between studies (as indicated by the authors), only the largest study was included in the current meta-analysis, in order to avoid double counting. Only baseline data were included from longitudinal study articles.

### Quality Assessment

Two researchers (KH and KS) independently assessed the risk of bias and methodological quality of the included studies using a modified version of the Newcastle-Ottawa Quality assessment Scale for case-control studies (22). Following assessments were added to the original scale: an evaluation of the sample size (i.e. a required sample size of minimum twenty patients), assessment of outcome consisting of an evaluation of the completeness of TRYCAT description on the one hand as well as lab procedures (including blinding) in order to guarantee reproducibility on the other hand) and an assessment of statistical reporting. Studies could obtain up to ten stars on three overall quality domains (i.e. selection, comparability, and outcome).

### Data Synthesis and Analysis

Demographic variables (age and gender), clinical assessments (mood state and symptom severity scores), and TRYCAT metabolite levels (means and standard deviations) were extracted from each study. Authors were contacted for additional information when data could not be extracted from the paper; this was received from four papers (13, 23–25). The Review Manager 5.3 (RevMan 5.3) computer program was used for performing the primary meta-analysis and subgroup analyses. The primary outcome measure was the standardized mean difference (SMD) in random effect models, represented in forest plot graphs (95% confidence interval). The presence of heterogeneity was assessed using Chi^2^ and its magnitude using I^2^ statistics. Potential effect modification by gender, age, and publication year was investigated by performing meta-regression analyses (Knapp-Hartung method, maximum likelihood) (26) in Comprehensive Meta-Analysis version 3 (CMA v3). For analyses with ten or more available studies, funnel plots and Egger’s tests were used to assess the presence of publication bias.

Subgroup analyses were performed to investigate the difference in TRYCAT levels for BP patients in a different mood state (depressed, manic). A minimum of three studies per subgroup was required in order to perform a subgroup analysis for each TRYCAT. A depressed state was defined as a major depressive episode as diagnosed by the RDC, DSM-(III, III-R, IV, IV-TR) or ICD-(9 or 10) criteria and/or defined as a minimum threshold of 17 or 18 on the Hamilton Rating Scale for Depression (HRSD-17) or 20 on the HRSD-24 (27). A manic state was defined as fulfilling the criteria of the RDC, DSM-(III, III-R, IV, IV-TR) or ICD-(9 or 10) ICD-10) criteria and/or as having a minimum threshold of 13 on the Young Mania Rating Scale (YMRS) (28), the most frequently used scale for assessment of manic symptoms. By means of a supplementary analysis, subgroup analyses were also performed to investigate the differences in effect size between high and low quality studies. The significance level was set at *p* < 0.05, the Benjamin-Hochberg procedure was applied for controlling false discovery rates (FDR) in meta-regression analyses.

## Results

### Study Selection

**Figure 2:**
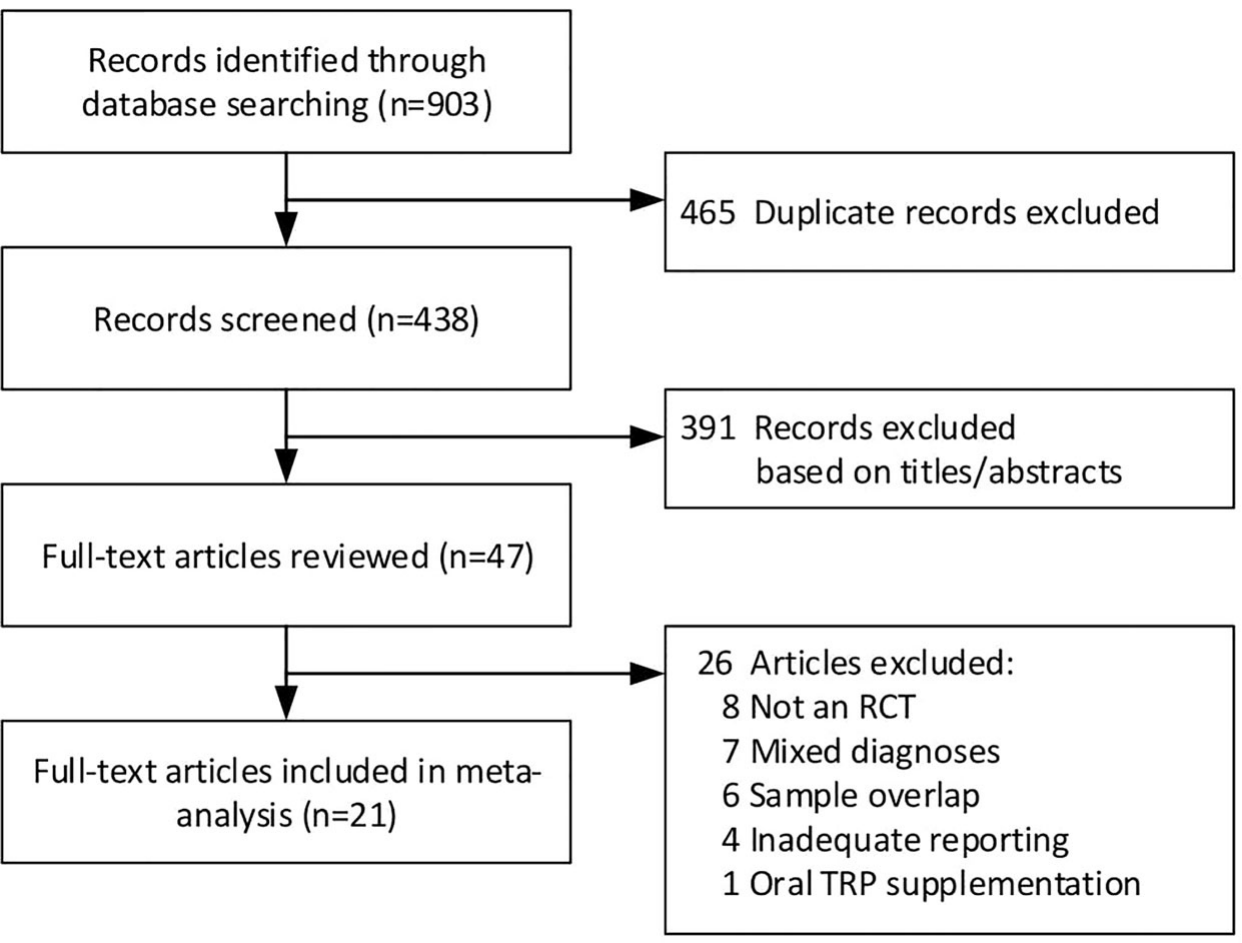
Flowchart.

The search strategy resulted in 903 hits and after deduplication 438 remained that were screened for relevance based on title and abstract. A final of 47 papers were read in full, of which 26 were excluded. The PRISMA Flow Diagram in **Figure 2** depicts the number of in- and excluded articles from each stage of screening. Four studies investigated TRYCATs in CSF (15, 29–31), sixteen in serum or plasma and one both in CSF and serum (13). Only one post-mortem study met inclusion criteria (32), but this study was excluded due to inadequate reporting. Of the twenty-one included papers in the meta-analysis, twelve had a cross-sectional design; nine a longitudinal design. **Table 1** presents the characteristics of the included studies. The analysis of TRP in CSF and five TRYCATs (TRP, KYN, KA, 3-HK, QA) in peripheral blood were included in the meta-analyses based on the minimal requirement of three studies for each meta-analysis.

**Table 1:** Characteristics of included studies.

Author, Year	No. of participants (P/HC)	Mean Age (SD)(P/HC)	%male (P/HC)	D/M/E	Sample Type	Longitudinal (yes/no)	TRP	KYN	3-HK	QA	KA
Ashcroft et al., 1973 (29)	13/26	NA	NA	D (6) M (7)	CSF	Yes	x				
Coppen et al., 1972 (30)	13/14	50.6 (4.09)/NA	23/50	D (10), M (3)	CSF	No	x				
Gerner et al., 1984 (31)	13/37	NA	NA	M (13)	CSF	No	x				
Olsson et al., 2012 (15)	55/23	39(14)/33.1(6.9)	38/100	E (55)	CSF	No					x
Sellgren et al., 2019 (13)	93/113	NA	NA	NA	CSF	No					x
**Total number of studies in CSF tissue (n = 5)**							**3**				**2**
Chiaroni et al., 1990 (33)	18/33	57.9(17.6)/39.5 (8.4)	36/42	D (18)	Plasma	Yes	x				
Hoekstra et al., 2006 (34)	32/20	47.2(14.6)/50.1 (13.5)	63/70	M (20) E (12)	Plasma	Yes	x				
Liu et al., 2018 (35)	20/23	30.5(5.1)/29.3 (5.9)	40/52	D (20)	Plasma	No	x	x		x	x
Moller and Amdisen, 1979 (36)	18/25	39.4 (14.1)/41.7 (12.4)	0/0	D (18)	Plasma	No	x				
Mukherjee et al., 2018 (17)	21/28	36.1 (11.3)/31.6 (10.3)	52/43	D (9), M (1), Mixed (10)*	Plasma	Yes	x	x			
Murata et al., 2020 (37)	43/26	42.3 (12.3)/39.6 (13.5)	48/52	D (43)	Serum	Yes	x	x			
Myint et al., 2007 (9, 14),	39/80	37.6 (11.6)/39.1 (8.8)	38/50	M (39)	Plasma	Yes	x	x			x
Olajossy et al., 23 (23)	11/48	44.7 (13.8)/35 (NA)	43/48	D (11)	Serum	Yes					x
Pan et al., 2018 (38)	30/40	35.8 (10.7)/36.8 (8.8)	43/55	NA	Plasma	No		x			
Platzer et al., 2017 (39)	68/93	44.9 (14.0)/38.9 (16.2)	62/39	E (68)	Serum	No	x	x	x		x
Poletti et al., 2018 (12)	22/15	46.5 (13.7)/27.2 (8.3)	36/43	D (22)	Serum	No	x	x	x		x
Poletti et al., 2019 (40)	72/36	48.0 (13.7)/43.9 (12.3)	40/36	D (55), M (17)	Plasma	No	x	x			
Savitz et al., 2015 (41)	63/48	38.8 (11.1)/32.6 (10.4)	19/40	D (63)	Serum	No	x	x	x	x	x
Sellgren et al., 2019 (13) (plasma)	163/114	34.0 (NR)/35.0 (NR)	39/46	NA	Plasma	No					x
Van den Ameele et al., 2020 (24)	67/34	43.1 (11.2)/42.7 (11.6)	42/46	D (35), M(32)	Plasma	Yes	x	x	x	x	x
Wurfel et al., 2017 (10)	53/92	40.2 (11.0)/32.3 (10.4)	30/36	D (15), M (25), Mixed (10)**	Serum	No	x	x	x	x	x
Zhou et al., 2018 (42)	16/6	37.8 (12.9)/31.6 (10.7)	69/58	D (16)	Serum	Yes	x	x			x
**Total number of studies in peripheral blood tissue (n = 17)**							**14**	**12**	**5**	**4**	**10**

*One patient did not have CARS-M data.

**Affective state missing from three patients.

SD, standard deviation; P, Patients; HC, healthy controls; D, Depressed; M, Manic; E, Euthymic; TRP, Tryptophan; KYN, kynurenine; 3-HK, 3-hydroxykynurenine; QA, Quinolinic acid; KA, Kynurenic Acid; CSF, cerebrospinal fluid; NA, not applicable. An overview of the total number of studies in CSF and peripheral blood are shown in bold.

Two CSF studies included both BD-D and BD-M patients (29, 30), one solely BD-M (31) and one solely euthymic BD patients (15). Eight serum/plasma studies included only BD-D (12, 23, 33, 35–37, 41, 42), one only BD-M (14), one only euthymic BD (39), two both BD-D and BD-M patients (24, 40), one study both BD-M and euthymic BD (34) and two studies BD-D, BD-M and BD-Mixed patients (10, 17). In the two remaining studies the mood state of BD patients was not specified (13, 38).

### Quality Assessment

The results of the quality assessment can be found in **Table 2**. The quality analysis showed an overall moderate methodological quality with 12 studies (57%) scoring half of the maximum score or more (5/10 or more). Eight studies (38%) had a sample size of less than twenty patients (12, 23, 29–31, 33, 36, 42). Only five studies recruited a matched control sample (13, 23, 24, 34, 38) and all but one study (10) reported unadjusted mean TRYCAT levels. Four studies reported that the laboratory technicians were blind for diagnose status (10, 14, 31, 41).

**Table 2:** Quality Analysis.

	SELECTION					COMPARABILITY	OUTCOME		TOTAL (max 10*)
	Case definition (max 1 *)	Sample size (max 1 *)	Selection of controls (max 1*)	Definition of controls (max 1*)	Outcome assessment (max 2*)	Comparability(max 2*)	Outcome Assessment (max 1*)	Statistical Analyses (max 1*)	
Ashcroft et al. (29)	0	0	0	0	A: * B: 0	0	0	0	1
Coppen et al. (30)	0	0	0	0	A: * B: 0	0	0	0	1
Gerner et al. (31)	0	0	0	0	A: * B: 0	0	*	0	2
Olsson et al. (15)	*	*	*	*	A:0 B:*	0	0	*	**6**
Sellgren et al. (13)	*	*	*	*	A:0 B:*	*	0	*	**7**
Chiaroni et al. (33)	0	0	0	0	A:* B: 0	0	0	*	2
Hoekstra et al. (34)	*	*	0	0	A: * B: 0	**	0	*	**6**
Liu et al. (35)	0	*	0	0	A:* B: 0	0	0	*	3
Moller and Amdisen (36)	0	0	*	0	A:* B: 0	0	0	0	2
Mukherjee et al. (17)	*	*	0	*	A:* B: 0	0	0	*	**5**
Murata et al. (37)	*	*	*	*	A:0 B: 0	0	0	0	4
Myint et al. (9, 14)	*	*	*	0	A:* B:*	0	*	0	**6**
Olajossy et al. (23)	0	0	0	0	A:0 B: 0	*	0	*	2
Pan et al. (38)	*	*	*	0	A:* B: 0	**	0	*	**7**
Platzer et al. (39)	*	*	0	*	A:* B: 0	0	0	*	**5**
Poletti et al. (12)	*	0	0	0	A:* B: 0	0	0	*	3
Poletti et al. (40)	*	*	0	*	A:* B: 0	0	0	*	**5**
Savitz et al. (41)	*	*	0	*	A:* B: 0	0	*	*	**6**
Van den Ameele et al. (24)	*	*	*	*	A:* B:*	**	0	*	**9**
Wurfel et al. (10)	0	*	*	*	A:* B:0	**	*	*	**8**
Zhou et al. (42)	*	0	*	*	A:* B:0	0	0	*	**5**

0: not satisfying minimum requirements (see **Supplement S1**).

* or ** in case of a maximum score of 2**: adequately satisfying minimum requirements.

### Central Levels of Kynurenine Metabolites

CSF levels of TRP did not significantly differ from healthy controls (n_studies_ = 3, n_patients_ = 39, SMD = - 0.43, *z* = 0.86, *p* = 0.39). There was considerable inter-study heterogeneity (I^2^: 83%, see **Supplementary Figure 4**). Only two studies investigated KA in CSF in BD. No CSF studies were found for KYN, 3-HK and QA in BD. Consequently, these four TRYCATs were not included in the meta-analysis.

### Peripheral Levels of Kynurenine Metabolites

Peripheral blood levels of TRP, KYN and KA were significantly lower in BD compared to healthy controls (TRP: n_studies_ = 14, n_patients_ = 552, SMD = -0.44, *z* = 4.94, *p* < 0.001; KYN: n_studies_ = 12, n_patients_ = 514, SMD = - 0.30, *z* = 3.21, *p* = 0.001; KA: n_studies_ = 10, n_patients_ = 522, SMD = - 0.45, *z* = 3.98, *p* <.001). Peripheral QA and 3-HK concentrations did not differ significantly between BD and healthy controls (QA: n_studies_ = 4, n_patients_ = 203, SMD = - 0.31, *z* = 1.37, *p* = 0.17; 3-HK: n_studies_ = 5, n_patients_ = 273, SMD = - 0.78, *z* = 0.54, *p* = 0.59). Inter-study heterogeneity was present for all TRYCATs with I^2^ ranging from 46 to 77%.

### Publication Bias

Funnel plots (of metabolites with a minimum of 10 available studies; TRP, KYN, KA in peripheral blood) are presented in **Supplementary Figures 1** to **3**. The funnel plot of KA shows a significant asymmetry, confirmed by the Egger’s test (shown in **Table 3**), which potentially indicates a publication bias in favor of research reporting lower KA levels in BD.

**Table 3:** Results of Egger’s tests for publication bias.

	Intercept	95% C.I.	*p (two-tailed)*
TRP_peripheral_	- 1.693	- 4.375 to 0.988	0.194
KYN_peripheral_	-1.946	- 4.799 to 0.907	0.160
KA_peripheral_	-2.812	-5.231 to -0.393	0.028*

TRP, tryptophan; KYN, kynurenine; KA, kynurenic acid; C.I., Confidence interval. *significant outcomes are shown in bold.

### Subgroup Analyses and Meta-Regression

Subgroup analyses in euthymic patients could not be reliably performed due to the scarcity of such studies, as there were only three studies including euthymic BD patients, of which one presented CSF levels. Subgroup effect by either depressed or manic mood state for TRP and KYN did not show effect modification (Chi^2^ test for subgroup differences were not significant, see **Supplementary Figures 11**–**12**). Subgroup analyses for KA, 3-HK and QA could not be performed since the minimum criterion of three studies in each subgroup was not fulfilled. The pooled effect estimate for TRP in the BD-M subgroup was slightly larger than that of the BD-D subgroup (BD-M: SMD = - 0.52; z = 2.32; *p* = 0.02; BD-D: SMD = - 0.43; z = 2.96; *p* = 0.003). The pooled effect estimates for KYN in BD-D and BD-M groups were comparable (BD-M: SMD = - 0.27; z = 1.98; *p* = 0.05; BD-D: SMD = - 0.38; z = 2.8; *p* = 0.005). Considerate within-subgroup heterogeneity remained, indicating that other unidentified factors likely affect TRYCAT levels in BD patients. By means of a supplementary analysis (**Supplementary Table 1**), we performed a subgroup analysis comparing effect sizes in high and low quality studies and this indicated a significant subgroup effect for KYN (*p* = 0.04) and KA (*p* = 0.04) with low quality studies showing larger effect sizes compared to high quality studies.

As demonstrated in the meta-regression analyses (see **Supplementary Table 2**), there was no effect modification for TRP, KYN and KA by age. The gender of the control group appeared to be a significant moderator of the effect in the studies comparing KA in BD and controls, yet this was no longer significant after correcting for false discovery rates. Meta-regressions could not be performed for 3-HK and QA due to the low number of studies (n = 5 and n = 4 respectively).

## Discussion

This meta-analysis summarizes the available evidence on a wide range of TRYCAT metabolites, representative for the whole kynurenine pathway, in BD patients compared to healthy controls. Patients with BD showed lower peripheral levels of TRP, KYN and KA compared to healthy controls. The levels of 3-HK and QA were not significantly different between healthy controls and BD. CSF levels of TRP showed no significant difference between BD and healthy controls, but this finding was based on only three studies.

Our results confirm that BD is associated with alterations in TRYCATs. However, these findings do not entirely correspond to the theoretically proposed hypotheses to explain the relationship between inflammation, kynurenine metabolism and BD. TRYCATs are assumed to act as inflammatory mediators and to cause neurodegeneration through neurotoxic effects (43), but the exact pathophysiological mechanism how TRYCATS influence BD symptoms and course remain unclear. The lower TRP levels in peripheral blood are consistent with the inefficient serotonin turnover in BD (14, 17), but our findings are not consistent with the theoretical hypothesis of an increased TRP breakdown, under low-grade inflammatory conditions (11), which would be expected to result in elevated KYN and KA levels. A plausible explanation for this inconsistency may be that a proposed microglial branch upregulation results in a reduced shunt towards the astrocytic branch, resulting in lower KYNA levels (44).

Our findings are in line with a recent meta-analysis by our group on TRYCAT alterations in schizophrenia spectrum disorder (SSD) which showed a partial downregulation of the kynurenine pathway (significantly lower levels of peripheral TRP in all SSD patients but especially in acute psychotic, younger patients and of peripheral KA and QA in symptomatic and/or older SSD patients (4). Accumulating evidence shows that acute psychotic exacerbations are associated with different immunological alterations than non-acute states (45, 46) and our group previously hypothesized differences in state (i.e. emerging during acute exacerbations) and trait immune markers (i.e. relatively unaltered throughout the disorder) in SSD (47), which could also be the case in BD.

However, it should be noted that peripheral, rather than central TRYCAT metabolites have been measured in most studies. An important question is to what extent CSF and plasma TRYCAT levels are correlated and how they differentially influence the pathophysiology of BD. TRP, KYN, and 3‐HK easily cross the blood brain barrier by active transport, but the brain uptake of QA and KA is limited to passive diffusion due to their polarity (48). Sellgren et al. (13) have demonstrated that peripheral KA levels do not mirror central levels in a large sample of BD and healthy controls. But other studies did show a correlation between QA and KA levels in serum and CSF in depressed patients with proven signs of inflammation levels (49, 50). A secondary issue concerns the binding capacity of TRP, KYN and KA to plasma proteins, such as albumin, but the exact result on peripheral values and blood-brain transport remains unclear (48). Third, the peripheral kynurenine pathway is regulated by immune markers, steroids and growth factors (51–53) which can also potentially affect peripheral levels.

All analyses of studies investigating the TRYCAT levels in peripheral blood showed substantial between-study heterogeneity, with effect sizes varying noticeably between studies. This suggests that a number of confounders and study-specific variables contribute to the effect size and, consequently, to the divergence in study results. We investigated the role of mood state (manic or depressed state) in subgroup analyses but this did not explain a significant proportion of the between-study variance. In a further attempt to reveal study-specific characteristics related with heterogeneity, meta-regression analyses were performed but these revealed no significant associations between TRYCAT levels and variables such as age, gender and publication year. It should be emphasized that other, not-investigated, factors could play a role in this heterogeneity. We can broadly categorize these factors into three domains: methodological, clinical and conceptual issues. Apart from differences in methodological quality between studies, differences in lab techniques could also lead to heterogeneous results. Although Liquid-Chromatography Mass Spectrometry is currently considered as golden standard and consequently the most commonly used method, other techniques have been used in studies such as High-Pressure Liquid Chromatography and Atomic Absorption Spectrophotometry***)***. Moreover, some TRYCAT metabolites (such as QA) have extremely low concentrations in peripheral blood tissue which tend to border the limits of the detection range of most of these methods, which may greatly affect reliability of some of these assessments. Several clinical factors are assumed to influence TRYCAT levels, the most of which is the use of psychotropic drugs. Several studies have demonstrated a moderating effect of anticonvulsants (e.g. valproate) on TRYCAT levels (24, 34) but there is a lack of large-scale studies. Moreover, age and duration of illness may similarly have an effect on TRYCAT changes, although the limited amount of available studies do not allow for proper analyses of these effects. Lastly, between-study heterogeneity could be a reflection of underlying genetic, phenotypical or diagnostic diversity of BD patients included in different studies (54). However, this heterogeneity, which may translate in differential impact on the TRYCAT pathway, has never been investigated in BD patient groups.

To our knowledge, this meta-analysis provides the most extensive summary of all available studies on a wide range of TRYCAT levels measured in CSF or serum/plasma in BD patients published to date. Compared to previously published meta-analyses (18, 19), we performed a broader literature search and provided a more complete analysis of the data by contacting authors for additional data on TRYCAT levels of BD subgroups. Other strengths of our study are the critical quality assessment of the included studies and the separate analysis of TRYCAT alterations in BD patients in a different mood (manic, depressed) resulting in a more nuanced picture of TRYCAT alterations in BD and adding evidence to the discussion on whether TRYCAT alterations should be considered as state or trait dependent changes. However, our results need to be interpreted in view of some limitations. Some analyses included only a small number of studies and the methodological quality of some studies was insufficient. The interpretation of our results is further limited by the differential use of psychopharmacological treatments between patients within and between studies as these are known to have a confounding influence on inflammatory mediators. The majority of the individual studies did not adjust the analysis for important confounders, such as age, gender, smoking status, duration of BD, (doses of) psychotropics, and symptom severity.

## Recommendations for Further Research

Peripheral TRYCAT levels were lower in BD than healthy controls, signaling a potential role in its pathophysiology. Our results indicate an overall lack of well-powered studies measuring downstream TRYCATs in BD. Future studies should aim to investigate intra-individual analyses of both peripheral and central TRYCAT levels, preferably in a longitudinal design, including patient groups stratified in symptomatic, medicated and age groups.

## Data Availability Statement

The original contributions presented in the study are included in the article/**Supplementary Material**. Further inquiries can be directed to the corresponding author.

## Author Contributions

KH and MM performed the literature search and statistical analyses. EG verified the analytical methods. KH and KS performed the quality analysis. All authors discussed the results and contributed to the final manuscript. All authors contributed to the article and approved the submitted version.

## Conflict of Interest

The authors declare that the research was conducted in the absence of any commercial or financial relationships that could be construed as a potential conflict of interest.
