# Supplementary material for: Tryptophan Catabolites in Bipolar Disorder: A Meta-Analysis
Source: Front Immunol. 2021 May 19;12:667179. doi: 10.3389/fimmu.2021.667179 (PMC8170319; doi:10.3389/fimmu.2021.667179)
Supplement: Supplementary file 1 [file DataSheet_1.docx]

Supplementary Material – Overview

**Supplement S1:** Search strategy p. 2 - 3

**Supplement S2:** Quality analysis p. 4 - 6

- Questionnaire
- Supplementary Table 1: Comparisons of the effect sizes in high quality studies versus lower quality studies

**Supplement S3:** Funnel Plots p. 7 - 8

- Supplementary Figure 1: Funnel Plot TRP
- Supplementary Figure 2: Funnel Plot KYN
- Supplementary Figure 3: Funnel Plot KA

**Supplement S4:** Forest Plots p. 9 - 10

- Supplementary Figure 4: Forest Plot TRP – CSF
- Supplementary Figure 6: Forest Plot TRP – Peripheral blood
- Supplementary Figure 7: Forest Plot KYN in peripheral blood
- Supplementary Figure 8: Forest Plot 3-HK in peripheral blood
- Supplementary Figure 9: Forest Plot QA in peripheral blood
- Supplementary Figure 9: Forest Plot KA in peripheral blood

**Supplement S5:** Subgroup analyses p. 11

- Supplementary Figure 10: Forest plot TRP – illness phase
- Supplementary Figure 11: Forest plot KYN – illness phase

**Supplement S6:** Meta-Regression p. 12

- Supplementary Table 2: Meta-Regression

# Search strategy

**Search strategy part 1: combination of three components (**TRYCATS **in** BD **in** BLOOD **AND/OR** CSF**)**

**Search strategy part 2: combination of two components (**TRYCATS **in** BD**)**

**(**(("Kynurenine"[Mesh] OR "Kynurenic Acid"[Mesh] OR "kynureni*"[tw] OR "3-hydroxykynurenine"[Supplementary Concept] OR "3-hydroxykynureni*"[tw] OR "hydroxykynureni*"[tw] OR "Picolinic Acids"[Mesh] OR "picolinic acid"[Supplementary Concept] OR "picolinic*"[tw] OR "Fusaric Acid"[Mesh] OR "Fusaric Acid"[tw] OR "Picloram"[tw] OR "Pyridoxic Acid"[tw] OR "Streptonigrin"[tw] OR "anthranilic acid"[Supplementary Concept] OR "anthranilic*"[tw] OR "xanthurenic acid"[Supplementary Concept] OR "xanthurenic*"[tw] OR "Tryptophan"[Mesh] OR "tryptophan"[tw] OR "Levotryptophan"[tw] OR "Ardeytropin"[tw] OR "Tryptan"[tw] OR "Optimax"[tw] OR "Lyphan"[tw] OR "5 Hydroxytryptophan"[tw] OR "Hydroxytryptophan"[tw] OR "Oxytryptophan"[tw] OR "5-HTP"[tw] OR "Oxitriptan"[tw] OR "Quinolinic Acid"[Mesh] OR "Quinolinic Acids"[Mesh] OR "QUINA"[tw] OR "Quinolinic*"[tw] OR "Quinolinate*"[tw]) AND ("Bipolar Disorder"[Mesh] OR "bipolar disorder*"[tw] OR "Bipolar Affective Psychosis"[tw] OR "Bipolar Depression"[tw] OR "Mania"[tw] OR "Manias"[tw] OR "Manic Depressive Psychosis"[tw] OR "Manic Disorder"[tw] OR "Manic Disorders"[tw] OR "Manic State"[tw] OR "Manic States"[tw] OR "Manic-Depressive Psychoses"[tw] OR "Manic-Depressive Psychosis"[tw] OR "manic"[tw] OR "bipolar affect*"[tw] OR "bipolar disease*"[tw] OR "euthym*"[tw] OR "Cyclothymic Disorder"[Mesh] OR "cyclothym*"[tw] OR "hypomani*"[tw]) AND ("blood"[Subheading] OR "blood"[tw] OR "blood"[Mesh] OR "plasma"[Mesh] OR "plasma"[tw] OR "serum"[Mesh] OR "serum"[tw] OR "sera"[tw] OR "CSF"[tw] OR "cerebrospinal fluid"[Subheading] OR "cerebrospinal fluid"[tw] OR "cerebrospinal fluid"[Mesh] OR "autopsy"[Mesh] OR "autops*"[tw] OR "postmortem"[tw] OR post-mortem[tw] OR "brain"[Mesh] OR "brain*"[tw])) **OR** (("Kynurenine"[majr] OR "Kynurenic Acid"[majr] OR "kynureni*"[ti] OR "3-hydroxykynurenine"[Supplementary Concept] OR "3-hydroxykynureni*"[ti] OR "hydroxykynureni*"[ti] OR "Picolinic Acids"[majr] OR "picolinic acid"[Supplementary Concept] OR "picolinic*"[ti] OR "Fusaric Acid"[majr] OR "Fusaric Acid "[ti] OR "Picloram"[ti] OR "Pyridoxic Acid"[ti] OR "Streptonigrin"[ti] OR "anthranilic acid"[Supplementary Concept] OR "anthranilic*"[ti] OR "xanthurenic acid"[Supplementary Concept] OR "xanthurenic*"[ti] OR "Tryptophan"[majr] OR "tryptophan"[ti] OR "Levotryptophan"[ti] OR "Ardeytropin"[ti] OR "Tryptan"[ti] OR "Optimax"[ti] OR "Lyphan"[ti] OR "5 Hydroxytryptophan"[ti] OR "Hydroxytryptophan"[ti] OR "Oxytryptophan"[ti] OR "5-HTP"[ti] OR "Oxitriptan"[ti] OR "Quinolinic Acid"[majr] OR "Quinolinic Acids"[majr] OR "QUINA"[ti] OR "Quinolinic*"[ti] OR "Quinolinate*"[ti]) AND ("Bipolar Disorder"[majr] OR "bipolar disorder*"[ti] OR "Bipolar Affective Psychosis"[ti] OR "Bipolar Depression"[ti] OR "Mania"[ti] OR "Manias"[ti] OR "Manic Depressive Psychosis"[ti] OR "Manic Disorder"[ti] OR "Manic Disorders"[ti] OR "Manic State"[ti] OR "Manic States"[ti] OR "Manic-Depressive Psychoses"[ti] OR "Manic-Depressive Psychosis"[ti] OR "manic"[ti] OR "bipolar affect*"[ti] OR "bipolar disease*"[ti] OR "euthym*"[ti] OR "Cyclothymic Disorder"[majr] OR "cyclothym*"[ti] OR "hypomani*"[ti]))**)** NOT ("Animals"[mesh] NOT "Humans"[mesh]) NOT (("Case Reports"[ptyp] OR "case report"[ti] OR "Review"[ptyp] OR "review"[ti]) NOT ("Clinical Study"[ptyp] OR "trial"[ti] OR "RCT"[ti]))

# Quality Analysis

## Questionnaire

**Based on:**The Newcastle-Ottawa Scale (NOS) for assessing the quality of nonrandomised studies in meta-analyses (adapted for case control studies)

GA Wells, B Shea, D O'Connell, J Peterson, V Welch, M Losos, P Tugwell,

**SELECTION (MAX 6*)**

1. **Case Definition (max 1*)**
   1. Clearly stated in- and exclusion criteria. Validated assessment tools (e.g., CIDI bipolar) are used to assess disease status. *
   2. Inadequate or not reported: not satisfying these requirements (in part), or not stated.
2. **Sample Size (max 1*)**
   1. Adequate: An appropriate (e.g. random sample) of minimum 20 eligible BD cases
   2. Inadequate or not reported: not satisfying these requirements (in part), or not stated.
3. **Selection of Controls (max 1*)**

- Adequate: the control series used in the study is derived from the same population as the cases and essentially could have become BD cases: e.g., community controls (i.e. same community as BD cases), hospital controls, within same community as cases. *
- Inadequate or not reported: not satisfying these requirements (in part), or not stated.

1. **Definition of Controls**
   1. Validated assessment tools (e.g., CIDI bipolar) are used to exclude a BD disease status.*
   2. Inadequate or not reported: not satisfying these requirements (in part), or not stated.
2. **Assessment of the outcome (a):**

- Adequate: description of TRYCAT level assessment is provided in detail, and according to internal standards, and is the same for cases and controls.*
- Inadequate or not reported: no description or insufficient description of TRYCAT level assessment, or assessment was not done similarly for cases and controls (or unclear)

1. **Assessment of the outcome (b):**

- Adequate: Adequate storage temperature for samples until analysis (e.g., at -70°/-80°). Lab technique is clearly represented. Measurement in duplicate.*
- Inadequate or not reported: not satisfying these requirements (in part), or not stated.

**COMPARABILITY (Max 2*)**

1. Either cases and controls must be matched in the design and/or confounders must be adjusted for in the analysis. (Statements of no differences between groups or that differences were not statistically significant are not sufficient for establishing comparability.)
   1. Adequate

- The study controls (or is matched) for the most important factor (age or sex) *
- The study control for any additional factor. *
  1. Inadequate or not reported: not satisfying these requirements (in part), or not stated.

**OUTCOME (Max 2 *)**

1. **Assessment of the outcome:**
   1. Adequate: The laboratory was blind for case- or control-status.*
   2. Inadequate or not reported: not satisfying these requirements (in part), or not stated.
2. **Statistical analyses:**
   1. Adequate: The statistical test used to analyze the data is clearly described and appropriate, and the measurement of the association is presented, including confidence intervals or SE, and/or the exact (non-rounded) probability level (p value). *
   2. Inadequate or not reported: not satisfying these requirements (in part), or not stated.

## Supplementary Table 1: Comparisons of the effect sizes in high quality studies versus lower quality studies

|  | No. of studies | No. of participants (P/HC) | SMD | Test for effect modification by study quality |
| --- | --- | --- | --- | --- |
| TRP |  |  |  | *p* = 0.85 |
| - High quality studies (> 4) - Low quality studies (≤ 4) | 9  5 | 431/437  121/122 | -0.45 [-0.59, -0.31]  -0.39 [-0.94, 0.15] |  |
| KYN |  |  |  | *p* = 0.04 |
| - High quality studies (> 4) - Low quality studies (≤ 4) | 9  3 | 429/457  85/64 | -0.24 [-0.42, -0.06]  -0.67 [-1.05, -0.29] |  |
| 3-HK |  |  |  | *p* = 0.26 |
| - High quality studies (> 4) - Low quality studies (≤ 4) | 4  1 | 251/267  22/15 | -1.23 [-2.56, 0.09]  0.6 [-2.27, -3.47] |  |
| QA |  |  |  | *NA* |
| - High quality studies (> 4) - Low quality studies (≤ 4) | 4  0 | 203/196  NA | -0.3 [-0.74, 0.14]  NA |  |
| KA |  |  |  | *p* = 0.04 |
| - High quality studies (> 4) - Low quality studies (≤ 4) | 6  4 | 453/461  522/553 | -0.33 [-0.57,-0.1]  -0.44 [-0.67,-0.23] |  |

**Abbreviations**. TRP = Tryptophan; KYN = kynurenine; 3-HK = 3-hydroxykynurenine; QA = Quinolinic acid; KA = Kynurenic Acid; SMD = Standard Mean Difference; NA = not applicable

# Funnel Plots

## Supplementary Figure 1: Funnel Plot TRP

## Supplementary Figure 2 Funnel Plot KYN

## Supplementary Figure 3 Funnel Plot KA

# Forest Plots

## Supplementary Figure 4: Forest Plot Tryptophan in CSF

## Supplementary Figure 5 Forest Plot TRP in peripheral blood


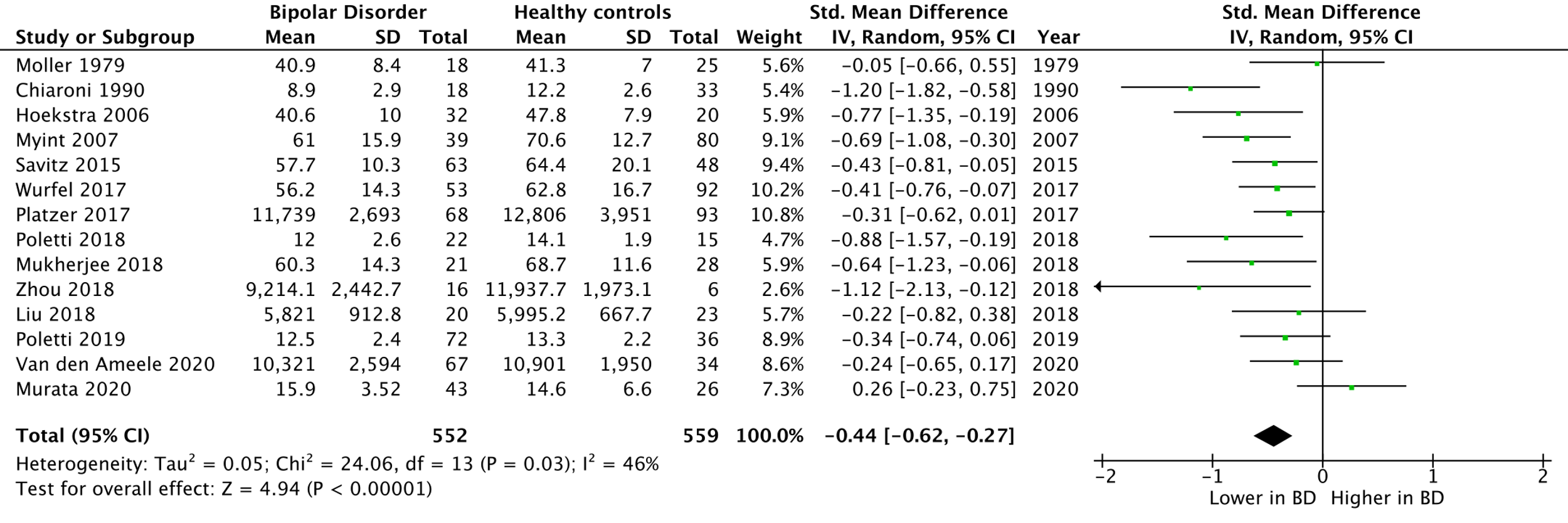


## Supplementary Figure 6: Forest Plot KYN in peripheral blood


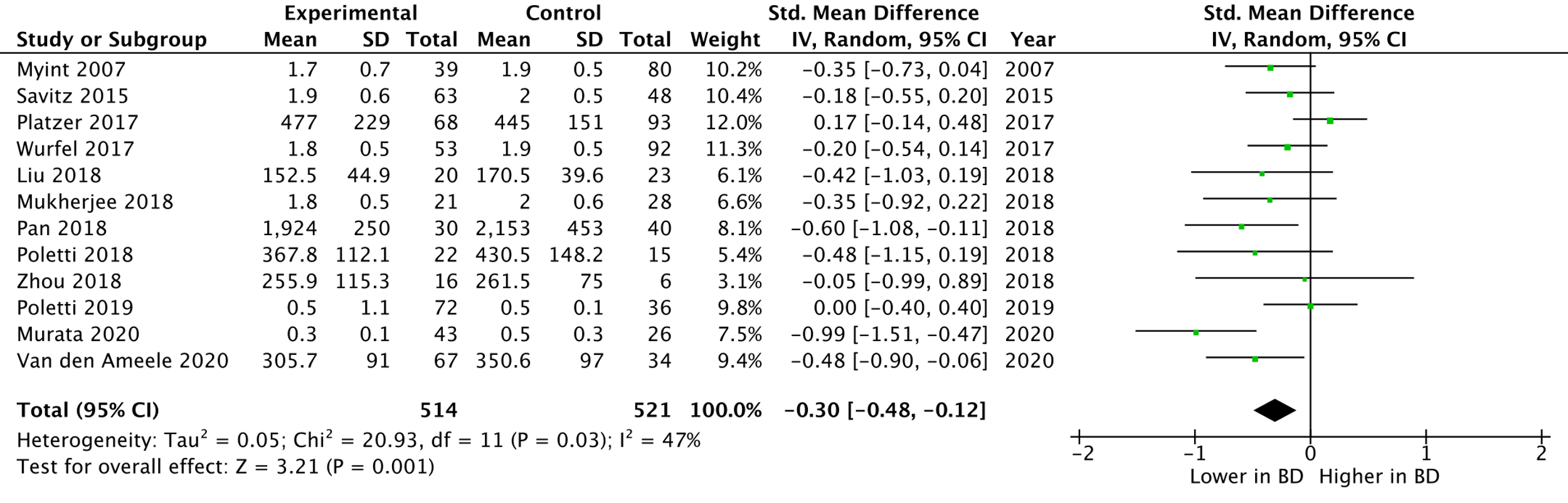


## Supplementary Figure 8: Forest Plot 3-HK in peripheral blood


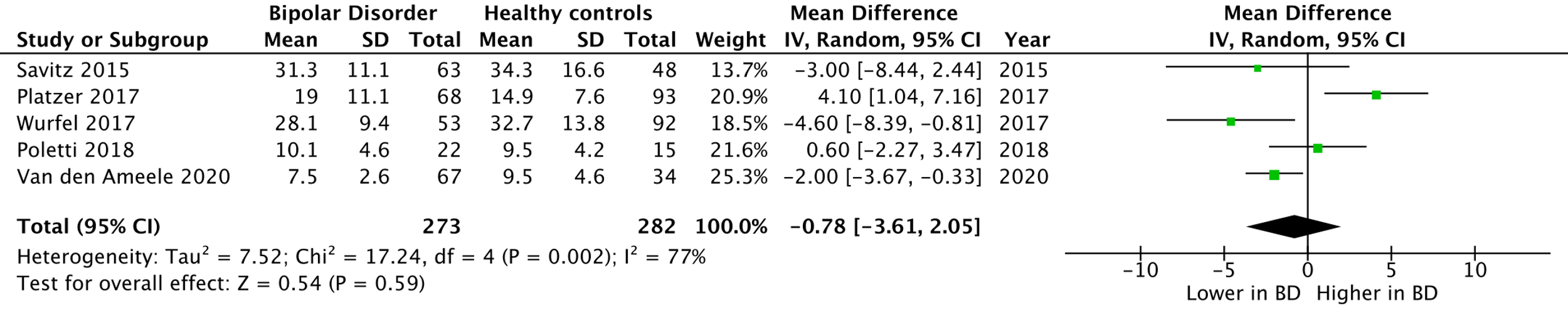


## Supplementary Figure 9: Forest Plot QA in peripheral blood

##
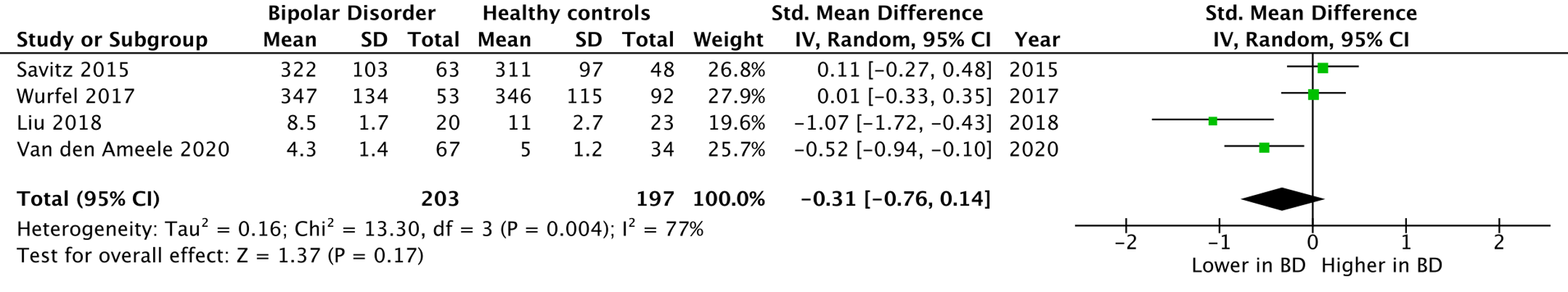


## Supplementary Figure 10: Forest Plot KA in peripheral blood


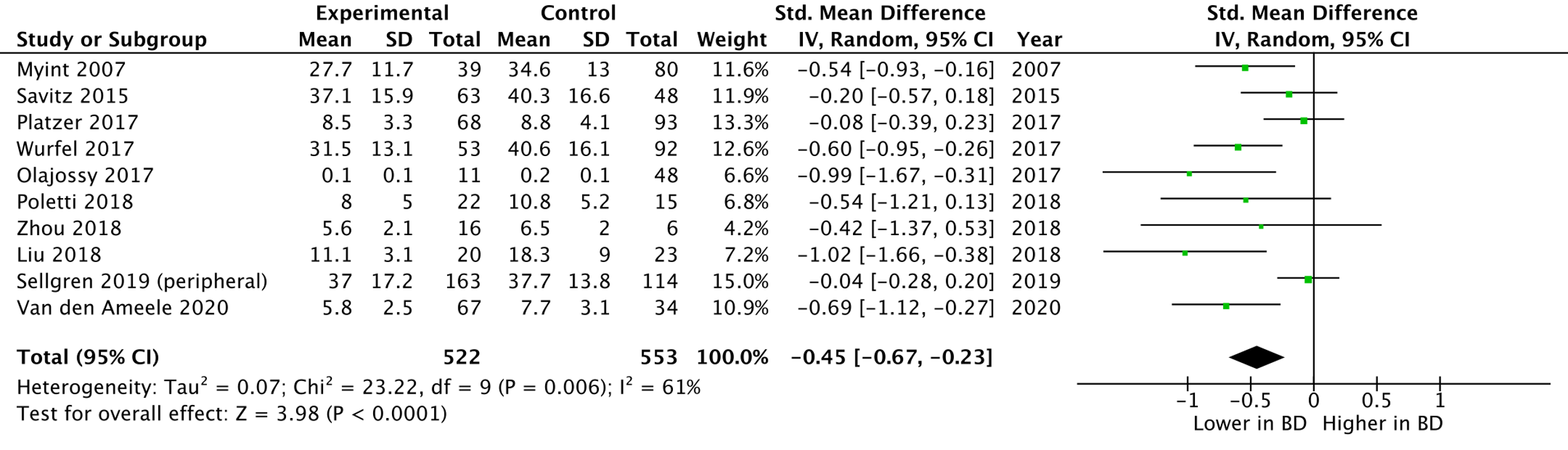


# Subgroup Analyses

## Supplementary Figure 11: Forest plot TRP – illness phase


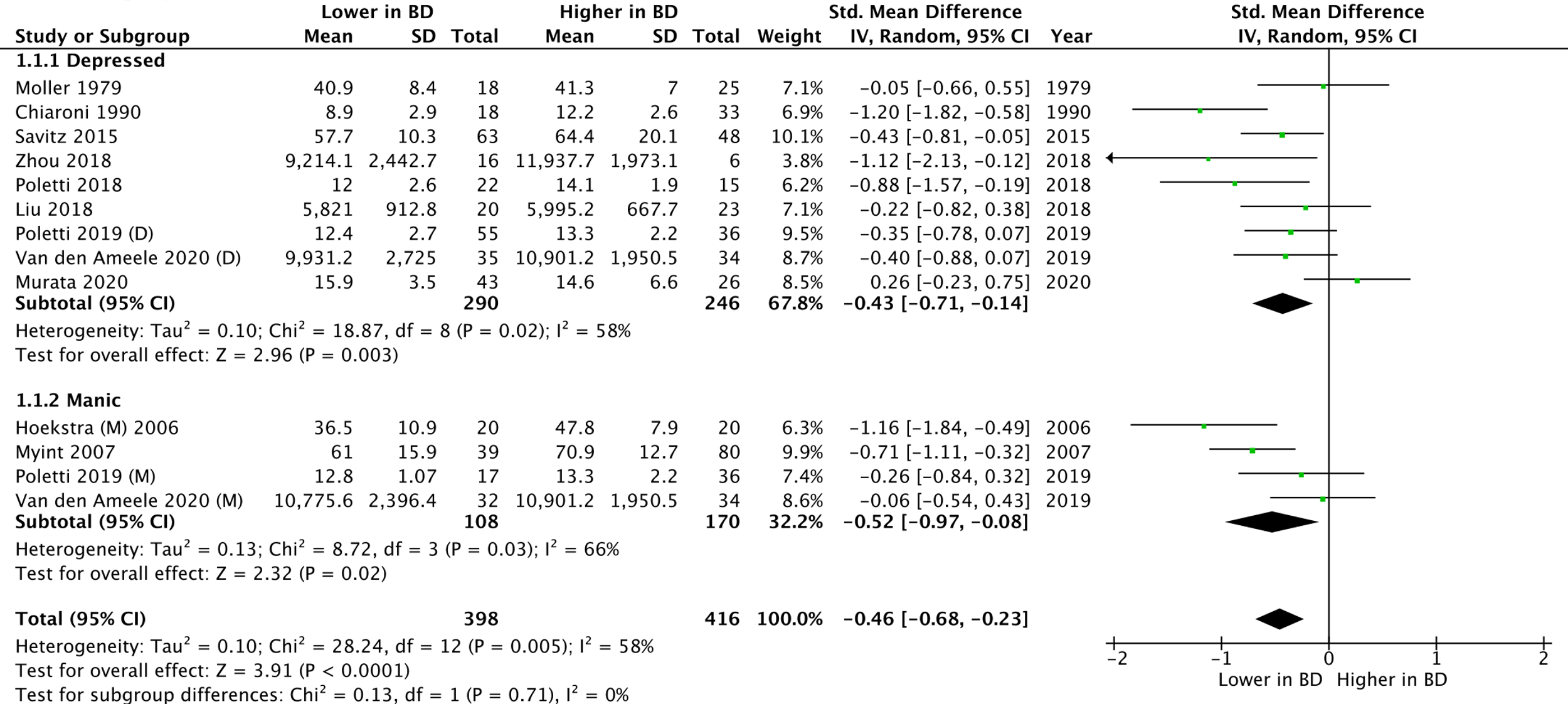


***Note.***

*Excluded from subgroup analyses: Wurfel (2017), Mukherjee (2018) (TRP levels from D, M, Mixed patients from not available), Platzer (2017) (Euthymic patients), Euthymic group from Hoekstra (2006)*

## Supplementary Figure 12: Forest plot KYN – illness phase

***
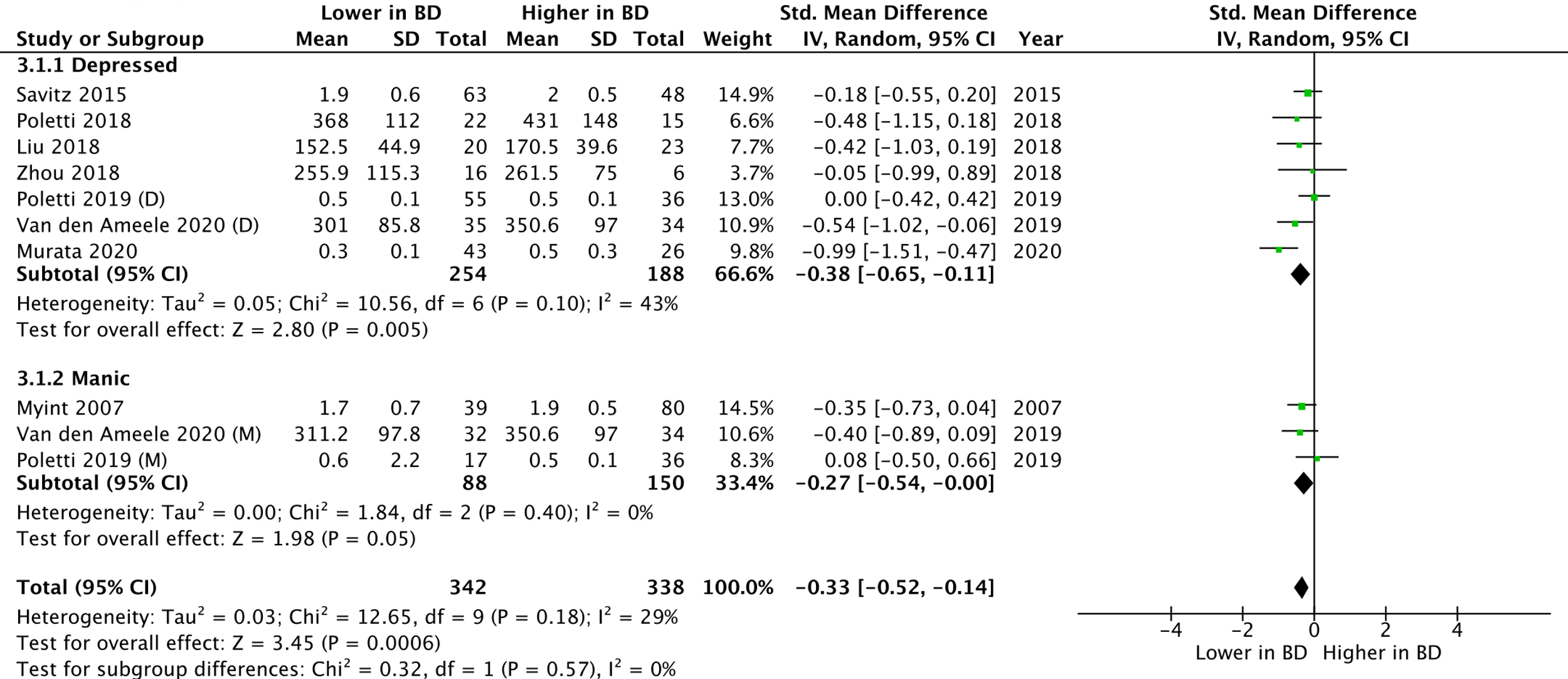
***

***Note.***

*Excluded from subgroup analyses: Wurfel (2017), Mukherjeen(2018) (TRP levels from D, M, Mixed patients from not available), Platzer (Euthymic patients)*

# Meta-Regression

*Supplementary Table 2: Meta regression analyses for Tryptophan (TRP), Kynurenine (KYN), and Kynurenic Acid (KA).*

|  | TRP  (n = 14) | KYN  (n=12) | KA  (n=10) |
| --- | --- | --- | --- |
| Age patients | t(1,12)=-1.13  *p* = 0.28 | t(1,10)=1.90  *p* = 0.09 | t(1,8)=.04  *p* = 0.97 |
| Age controls | t(1,12)=.59  *p* = 0.56 | t(1,10)=.84  *p* = 0.42 | t(1,8)=.46  *p* = 0.66 |
| Gender patients | t(1,12)=-.57  *p* = 0.58 | t(1,10)=.96  *p* = 0.36 | (1,8)=.43  *p* = 0.68 |
| Gender controls | t(1,12)=-.94  *p* = 0.37 | **t(1,10)=.-2.44**  ***p* = 0 .035** | t(1,8)=-1.08  *p* = 0.31 |
| Publication year | t(1,12)=.91  *p* = 0.38 | t(1,10)=-.20  *p* = 0.85 | t(1,8)=.31  *p* = 0.77 |

**Abbreviations**. TRP = Tryptophan; KYN = kynurenine; KA = Kynurenic Acid.
